# Supplementary material for: Unidirectional ray polaritons in twisted asymmetric stacks
Source: Nat Commun. 2024 Oct 19;15:9042. doi: 10.1038/s41467-024-52750-3 (PMC11490623; doi:10.1038/s41467-024-52750-3)
Supplement: Supplementary file 1 — Supplementary Information [file 41467_2024_52750_MOESM1_ESM.pdf]

SUPPLEMENTARY INFORMATION:

UNIDIRECTIONAL RAY POLARITONS IN TWISTED ASYMMETRIC STACKS

J. Álvarez-Cuervo[1,2,†], M. Obst[3,4,†], S. Dixit[5,†], G. Carini[6], A. I. F. Tresguerres-Mata[1,2], C. Lanza[1,2], E. Terán-García[1,2], G. Álvarez-Pérez[1,2,6,7], L.F. Álvarez-Tomillo[1,2], K. Diaz-Granados[5], R. Kowalski[5], A. S. Senerath[5], N. S. Mueller[6], L. Herrer[8], J.M. De Teresa[8], S. Wasserroth[6], J. M. Klopf[9], T. Beechem[10], M. Wolf[6], L.M. Eng[3,4], T.G. Folland[11], A. Tarazaga Martín-Luengo[1,2], J. Martín-Sánchez[1,2], S.C. Kehr[3,4*], A.Y. Nikitin[12,13,*], J.D. Caldwell[5,*], P. Alonso-González[1,2,*], A. Paarmann[6,*]

[1]Department of Physics, University of Oviedo, Oviedo 33006, Spain.
[2]Center of Research on Nanomaterials and Nanotechnology (CINN), CSIC-Universidad de Oviedo, El Entrego 33940, Spain.
[3]Institute of Applied Physics, TUD Dresden University of Technology, Dresden, Germany.
[4]Würzburg-Dresden Cluster of Excellence - EXC 2147 (ct.qmat), Dresden 01062, Germany
[5]Vanderbilt University, Nashville, TN, USA.
[6]Fritz Haber Institute of the Max Planck Society, Berlin, Germany.
[7]Center for Biomolecular Nanotechnologies, Istituto Italiano di Tecnologia, Via Barsanti 14, Arnesano, 73010, Italy
[8]Instituto de Nanociencia y Materiales de Aragón (INMA), CSIC-Universidad de Zaragoza, Zaragoza 50009, Spain.
[9]Institute of Radiation Physics, Helmholtz-Zentrum Dresden-Rossendorf, Dresden, Germany.
[10]Purdue University and Birck Nanotechnology Center, West Lafayette, IN, USA.
[11]University of Iowa, Iowa City, IA, USA.
[12]Donostia International Physics Center (DIPC), Donostia-San Sebastián 20018, Spain.
[13]IKERBASQUE, Basque Foundation for Science, Bilbao 48013, Spain.
*Correspondence to:
susanne.kehr@tu-dresden.de, alexey@dipc.org, josh.caldwell@vanderbilt.edu, pabloalonso@uniovi.es, alexander.paarmann@fhi-berlin.mpg.de
†These authors contributed equally to this work

Supplementary Note 1. Dispersion relation under high-q approximation of surface polaritons in a thin layer embedded by anisotropic media

Supplementary Note 2. Near-field s-SNOM visualization of PhPs propagating in asymmetric homostructures

Supplementary Note 3. Propagation of URPs in a twisted (θ=30°) asymmetric homostructure as a function of illumination frequency

Supplementary Note 4. Relation between the polaritonic IFCs of a symmetric bilayer and an asymmetric twisted homostructure

Supplementary Note 5. Analysis of the polaritonic propagation in a twisted ($\theta = 60^{o}$) homostructure as a function of thickness disparity

Supplementary Note 6. Analysis of the URPs response as a function of twist angle and illumination frequency in the homostructure stack

Supplementary Note 7. Determining the twist angle between a-axes of monoclinic β-Ga_2O_3 and α-MoO_3 using polarization-resolved Raman spectroscopy

Supplementary Note 8. Near-field s-SNOM visualization of PhPs propagating in asymmetric heterostructures

Supplementary Note 9. Explanation of the ray appearance as a function of the substrate permittivity values

Supplementary Note 10. Impact of an anisotropic substrate on the polariton propagation

Supplementary Note 11. Evolution of the β-Ga_2O_3 permittivity in the explored frequency region

Supplementary Note 12. Influence of the β-Ga_2O_3 permittivity on the ray-like propagation in α-MoO_3

Supplementary Note 13. Analysis of the URPs response as a function of twist angle and illumination frequency in the heterostructure stack

Supplementary Note 1. Dispersion relation under high-q approximation of surface polaritons in a thin layer embedded by anisotropic media

Under high-q approximation the dispersion equation of surface polaritons in a twisted bilayer heterostructure embedded by two isotropic media is[1]:

$$\begin{aligned} &-\tan(\xi_1)\tan(\xi_2)\,[\Sigma_s\Sigma_2^2+\Sigma_1^2\Sigma_S]+(-i)\,\tan(\xi_1)\,\Sigma_2[\Sigma_s\Sigma_S+\Sigma_1^2] \\ &+(-i)\,\tan(\xi_2)\,\Sigma_1[\Sigma_s\Sigma_S+\Sigma_2^2]+\Sigma_1\Sigma_2[\Sigma_s+\Sigma_S]=0 \end{aligned}$$

$$\xi_{\mathrm{i}}\equiv q_{\mathrm{iz}}k_0d_{\mathrm{i}} \qquad \Sigma_i\equiv\varepsilon_{iz}q_{iz}, \qquad i=1,2 \qquad \Sigma_{s,S}=i\varepsilon_{s,S}q \qquad \text{Eq. S1}$$

$$q_{\mathrm{iz}}=\sqrt{-\frac{1}{\varepsilon_{iz}}\left(\varepsilon_{ix}q_x^2+\varepsilon_{iy}q_y^2+2\varepsilon_{ixy}q_xq_y\right)}$$

where the indexes $i=s,1,2,S$ correspond to the substrate, bottom and top layers and the superstrate, respectively, and the terms ε_{ij}, $j=x,y,xy,z$ are the values of the permittivity tensor of the i-th layer.

By considering explicitly that one of the layers is very thick, i.e. $d_i\to\infty$, we can derive the analytic expression for surface polaritons in a single biaxial layer placed over an anisotropic substrate as follows:

Applying that $\tan(a+bi)\xrightarrow{\mathrm{b}\to\pm\infty}\pm i$, the condition $d_1\to\infty$ implies

$$\tan(\xi_1)=\tan(q_{1z}k_0d_1)\xrightarrow{d_1\to\infty} i\,\mathrm{sgn}(\mathrm{Im}(q_{1z}))$$

without the loss of generality, we can assume $\mathrm{Im}(q_{iz}) > 0$ so

$$\tan(\xi_i) = i$$

Substituting this condition in Equation (S1), we find:

$$-i\tan(\xi_2)\,[\Sigma_s\Sigma_2^2 + \Sigma_1^2\Sigma_S] + (-i)\,i\,\Sigma_2[\Sigma_s\Sigma_S + \Sigma_1^2] + (-i)\,\tan(\xi_2)\,\Sigma_1[\Sigma_s\Sigma_S + \Sigma_2^2] + \Sigma_1\Sigma_2[\Sigma_s + \Sigma_S] = 0$$

Regrouping the terms:

$$-i\tan(\xi_2)\,[\Sigma_s\Sigma_2^2 + \Sigma_1^2\Sigma_S + \Sigma_s\Sigma_1\Sigma_S + \Sigma_1\Sigma_2^2] + \Sigma_2[\Sigma_s\Sigma_S + \Sigma_1^2 + \Sigma_1\Sigma_s + \Sigma_1\Sigma_S] = 0$$

From which we can decouple two solutions:

$$[\Sigma_s + \Sigma_1]\left[(-i)\,\tan(\xi_2)\,[\Sigma_1\Sigma_S + \Sigma_2^2] + \Sigma_2[\Sigma_1 + \Sigma_S]\right] = 0 \qquad \text{Eq. S2}$$

The first term in Equation (S2) represents the dispersion of surface polaritons at the interface between two semi-infinite media under the high-q approximation[2]. The second oplne corresponds to the dispersion of polaritons in a single biaxial layer placed between an isotropic superstrate and an anisotropic substrate. By reducing the right-hand term of Equation (S2) we obtain a generalization of the well-known expression of the single layer dispersion[2]:

$$\tan(\xi_2) = -i\frac{\Sigma_2[\Sigma_1 + \Sigma_S]}{\Sigma_1\Sigma_S + \Sigma_2^2} = -i\frac{\frac{\Sigma_1}{\Sigma_2} + \frac{\Sigma_S}{\Sigma_2}}{1 + \frac{\Sigma_1\Sigma_S}{\Sigma_2^2}} = \frac{(-i)\frac{\Sigma_1}{\Sigma_2} + (-i)\frac{\Sigma_S}{\Sigma_2}}{1 - i^2\frac{\Sigma_1\Sigma_S}{\Sigma_2^2}}$$

Applying the relation $\mathrm{atan}\left(\frac{x+y}{1-xy}\right) = \mathrm{atan}\,x + \mathrm{atan}\,y$ we obtain:

$$\xi_2 = \mathrm{atan}\left(-i\frac{\Sigma_1}{\Sigma_2}\right) + \mathrm{atan}\left(-i\frac{\Sigma_S}{\Sigma_2}\right) + \pi l, \qquad l = 0,1,\ldots$$

Substituting the parameters $\xi_2 = q_{2z}k_0d_2,\ \Sigma_i = \varepsilon_{iz}q_{iz},\ i = 1,2$ and $\Sigma_S = i\varepsilon_S q$, we obtain:

$$q_{2z}k_0d_2 = \mathrm{atan}\left(-i\frac{\varepsilon_{1z}q_{1z}}{\varepsilon_{2z}q_{2z}}\right) + \mathrm{atan}\left(-i\frac{i\varepsilon_S q}{\varepsilon_{2z}q_{2z}}\right) + \pi l, \qquad l = 0,1,\ldots \qquad \text{Eq. S3}$$

Finally, we define the following parameter for each layer[2]:

$$\rho_i = i\sqrt{\frac{\varepsilon_{iz}q^2}{\varepsilon_{ix}q_x^2 + \varepsilon_{iy}q_y^2 + 2\varepsilon_{ixy}q_xq_y}} = i\sqrt{\frac{\varepsilon_{iz}}{\varepsilon_{ix}\cos^2\varphi + \varepsilon_{iy}\sin^2\varphi + 2\varepsilon_{ixy}\cos\varphi\sin\varphi}} \Longrightarrow$$

$$\Longrightarrow q_{iz} = -\frac{q}{\rho_i(\varphi)}$$

Substituting these parameters in Equation (S3) we obtain the explicit dispersion for surface polaritons under the high-q approximation in a single biaxial layer placed over an anisotropic substrate:

$$q = \frac{\rho_2}{k_0d_2}\left[\mathrm{atan}\left(i\frac{\varepsilon_{1z}\rho_2}{\varepsilon_{2z}\rho_1}\right) + \mathrm{atan}\left(\frac{\varepsilon_S\rho_2}{\varepsilon_{2z}}\right) + \pi l\right] \qquad l = 0,1,\ldots$$

Following the same procedure, we can derive the polariton dispersion for a single biaxial layer surrounded by two anisotropic semi-infinite media:

$$q = \frac{\rho}{k_0 d}\left[\operatorname{atan}\left(i\frac{\varepsilon_{sz}\rho}{\varepsilon_z\rho_s}\right) + \operatorname{atan}\left(i\frac{\varepsilon_{Sz}\rho}{\varepsilon_z\rho_S}\right) + \pi l\right] \qquad l = 0,1,\ldots \qquad \text{Eq. S4}$$

Supplementary Note 2. Near-field s-SNOM visualization of PhPs propagating in asymmetric homostructures

Supplementary Fig. 1 shows s-SNOM images of homostructures stacks with twist angles $\theta = 15^{o}$ (Supplementary Fig. 1a-e), 30° (Supplementary Fig. 1f-k) and 60° (Supplementary Fig. 1l-o). For each stack several illumination frequencies from $\omega = 870\ \mathrm{cm}^{-1}$ to $\omega = 920\ \mathrm{cm}^{-1}$ in $10\ \mathrm{cm}^{-1}$ steps were measured. As mentioned in the main text, a 200-nm diameter hole was fabricated into the three stacks to efficiently launch PhPs. The row of images corresponding to a twist angle of 30° completes the experimental images shown in Fig. 2. We can clearly see how the URPs appear consistently for the different frequencies. Additionally, the direction of the URPs increases gradually with increasing frequency while the number of fringes reduces. Interestingly, the near-field images corresponding to a twist angle of 15° (top row) also exhibit URPs, which show an almost constant phase. Similar to the case of a twist angle of 30°, the direction of propagation of these URPs increases with increasing frequency. For the case of the stack with a twist angle of 60° (Supplementary Fig. 1m-o), we observe "pinwheel" propagation patterns, which also evolve towards elliptic at $\omega = 880\ \mathrm{cm}^{-1}$. This finding remarks the unique properties of these asymmetric stacks and the diversity of the PhPs behaviors observed within them. Supplementary Fig. 2 shows full-wave numerical simulations corroborating these experimental results.

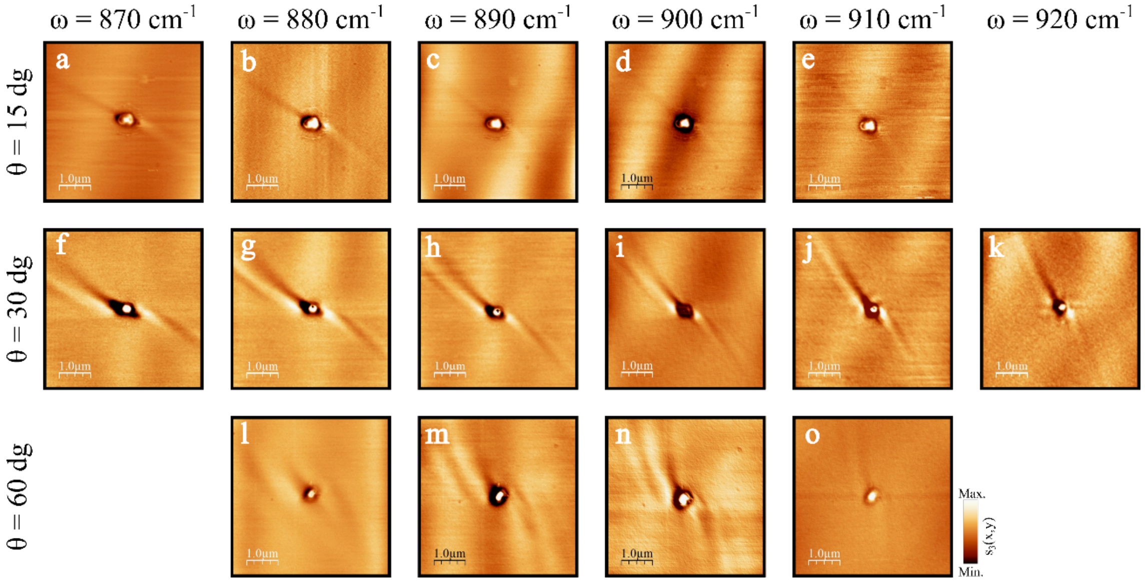

Supplementary Figure 1: Observation of unidirectional ray polaritons (URPs) in twisted asymmetric homostructures. a-o. Near-field s-SNOM (amplitude) images of stacks made of a thin ($d_{top} = 80$ nm) and a thick α-MoO_3 layer ($d_{bot} = 3$ µm) with a twist angle between them $\theta = 15^{o}$ (**a-e**), 30° (**f-k**) and 60° (**l-o**) at illuminating frequencies ranging from $\omega = 870\ \mathrm{cm}^{-1}$ to $920\ \mathrm{cm}^{-1}$. A 200-nm diameter hole allows efficient launching of the PhPs. The in-plane x (resp. y) direction is aligned to the [100] (resp. [001]) crystal direction of the top α-MoO_3 layer.

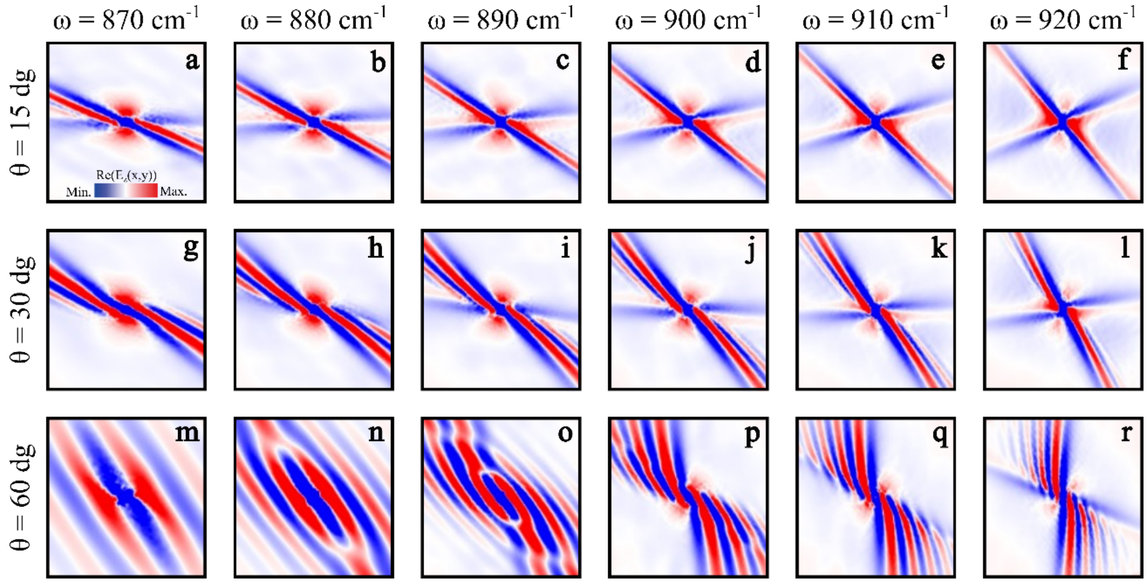

Supplementary Figure 2: Numerical simulations of unidirectional ray polaritons in twisted asymmetric homostructures. a-o. Simulated electric field $\mathrm{Re}(E_z)$ produced by a point electric dipole placed over a twisted structure made of a thin ($d_{top} = 80$ nm) and a thick α-MoO_3 layer ($d_{bot} = 3$ µm) with twist angles $\theta = 15^{o}$ (**a-f**), 30° (**g-l**) and 60° (**m-p**) at illuminating frequencies ranging from $\omega = 870\ \mathrm{cm}^{-1}$ to $920\ \mathrm{cm}^{-1}$. The in-plane x (resp. y) direction is aligned with the [100] (resp. [001]) crystal direction of the top α-MoO_3 layer.

Supplementary Note 3. Propagation of URPs in a twisted (θ=30°) asymmetric homostructure as a function of illumination frequency

In the following we theoretically explain the variation of the propagation direction of URPs as a function of illuminating frequency. We start showing the variation calculated numerically for a twisted ($\theta = 30^{o}$) homostructure (Supplementary Fig. 3a) for three different frequencies: $\omega = 880\ \mathrm{cm}^{-1}$, $900\ \mathrm{cm}^{-1}$ and $920\ \mathrm{cm}^{-1}$ (Supplementary Fig. 3c-e, respectively). We also represent the IFCs of the URPs (Supplementary Fig. 3f-h) by performing 2D-FFTs to the simulated images. They result in a single intense branch that reflects the unidirectional propagation of URPs with wavevectors almost completely perpendicular to this direction. As in Fig. 3 of the main manuscript, we show that the IFCs of the homostructure tend to the dispersion of the symmetric bilayer stack (white dashed curved in Supplementary Fig. 3f-h) for the frequencies analysed. In every case, the IFC of the homostructure is not completely straight but has some curvature that approaches the high k values of the symmetric bilayer case. Note that the k values in the IFC of the symmetric bilayer increase with frequency. Consequently, for $\omega = 880\ \mathrm{cm}^{-1}$ the IFC of the homostructure gets curved earlier generating more visible polaritonic fringes in the real-space images. In contrast, for $\omega = 920\ \mathrm{cm}^{-1}$ the IFC can be more straight generating less visible polaritonic fringes.

On the other hand, the propagation direction of URPs is mainly defined by one of the two excited ray modes in an independent thick α-MoO_3 layer (Supplementary Fig. 3b), which appear symmetrically (Supplementary Fig. 3i-k) with respect to the [100] α-MoO_3 axis (fixed, in this case, to an in-plane angle of 30°). Remarkably, the open angle of the symmetric ray pattern with respect to the [100] α-MoO_3 axis increases with increasing frequency, being consistent with the

frequency evolution of the propagation direction of URPs. In particular, the direction of propagation of the enhanced and suppressed rays of the twisted homostructure align with one of each ray present in the thick layer case. This is even clearer comparing the FFTs extracted for each case (Supplementary Fig. 3f-h and Supplementary Fig. 3l-n).

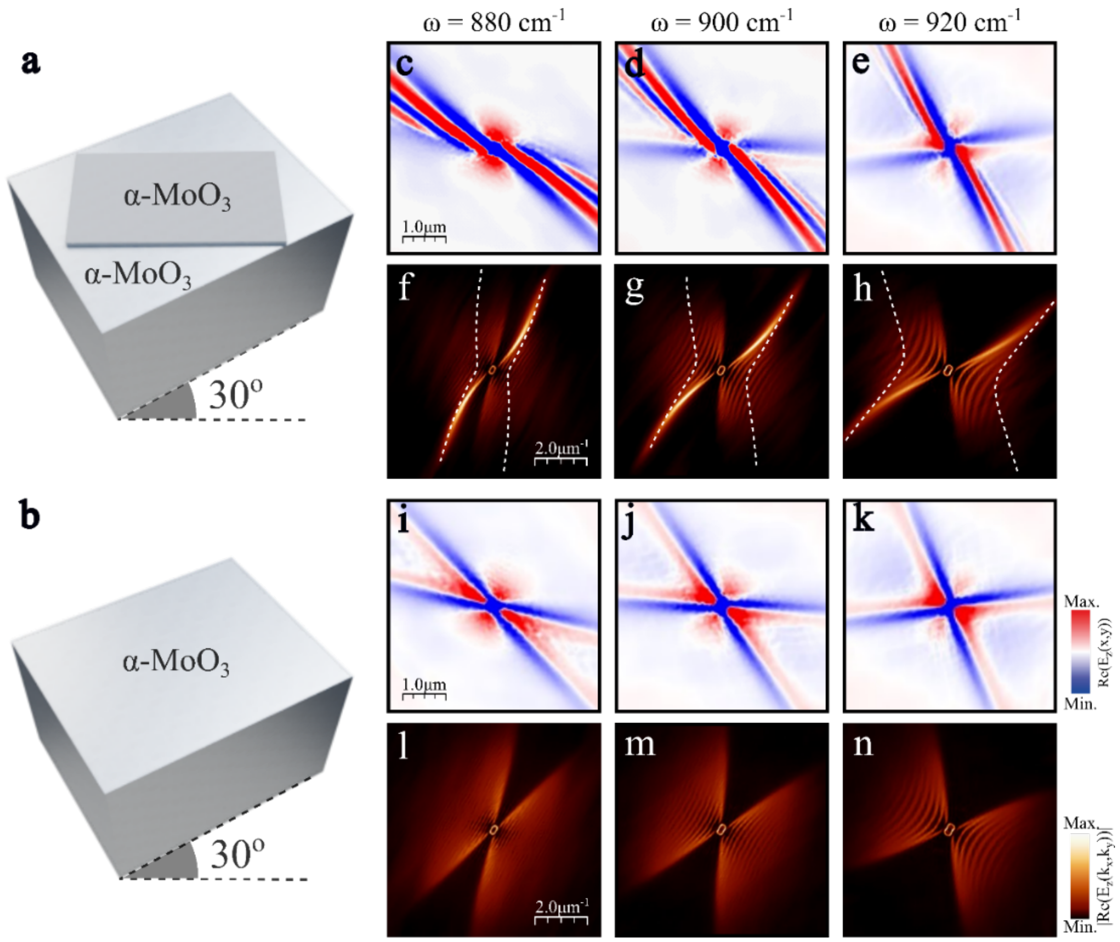

Supplementary Figure 3: Analysis of the unidirectional propagation of ray polaritons in a twisted ($\theta = 30^{o}$) asymmetric homostructure as a function of incident frequency. a-b. Schematic of the two systems under study: **a.** an 80 nm-thin α-MoO_3 layer placed over a 3 μm-thick α-MoO_3 layer (homostructure) and **b.** a single 3 μm-thick α-MoO_3 layer. In both cases, the twist angle is $\theta = 30^{o}$ while the illumination frequency varies from $\omega = 880\ \mathrm{cm}^{-1}, 900\ \mathrm{cm}^{-1}$ to $920\ \mathrm{cm}^{-1}$. **c-e, i-k.** Real-space electric field $\mathrm{Re}(E_z)$ launched by a point dipole source located above the homostructure (**c-e**) and the single thick layer (**i-k**). The illumination frequencies are $\omega = 880\ \mathrm{cm}^{-1}$ (**c,i**), $900\ \mathrm{cm}^{-1}$ (**d,j**) and $920\ \mathrm{cm}^{-1}$ (**e,k**). **f-h, l-n.** IFCs obtained by performing the 2D-FFTs of the near-field images in **c-e**,**i-k**, respectively. White dash curves in **f-h** correspond to the analytic IFC of the symmetric bilayer case obtained using Equation (S1).

Supplementary Note 4. Relation between the polaritonic IFCs of a symmetric bilayer and an asymmetric twisted homostructure

In the following, we explain the relation between the IFCs of a symmetric bilayer and a twisted homostructure through an analytical perspective. In particular, we study the influence of the thickness of the bottom layer d_1 in the analytic expression for the dispersion of surface polaritons in a bilayer (Equation (S1)). As explained in section S1, the thickness of the bottom layer only appears in the term $\tan(\xi_1)$. If d_1 is sufficiently large, mimicking a biaxial substrate, this term fulfils $\mathrm{Im}(\xi_1) \to \infty$ which translates into the condition $\tan(\xi_1) = i$. This allows to

obtain the dispersion of a single layer placed over an anisotropic substrate, as expressed in Equation (S4). However, even for a finite thickness of the bottom layer, as in the symmetric bilayer case, it is possible to satisfy this condition. Note that the term $\xi_1 = q_{1z}k_0d_1$ is proportional to the PhP momentum q. Then, if the polariton is sufficiently confined, i.e. q is sufficiently high, the ξ_1 term can be large enough to obtain $\tan(\xi_1) = i$. This means that, even without increasing the thickness of the bottom layer, this layer acts as an effective semi-infinite medium. Consequently, the IFCs of the symmetric bilayer and the asymmetric homostructure can fit for high k values.

To corroborate the validity of this argumentation, we analyse in Supplementary Fig. 4a the IFC of a symmetric twisted bilayer ($\theta = 30^{o}$) made of two 80 nm-thin α-MoO_3 layers at an illumination frequency $\omega = 900\,\mathrm{cm}^{-1}$, where the colorplots are defined by the value $\mathrm{Im}(\tan(\xi_1))$. Note that the brightest part of the IFC corresponds to angular values where $\mathrm{Im}(\tan(\xi_1)) = 1$ (Supplementary Fig. 4b) and $\mathrm{Re}(\tan(\xi_1)) = 0$ (Supplementary Fig. 4c). Thus, this part of the IFC of the twisted symmetric bilayer satisfies the condition $\tan\xi_1 = i$. Consequently, at this angular range the 80 nm-bottom layer can be considered as an effective semi-infinite medium explaining why its IFC fits the IFC of the twisted asymmetric homostructure.

To understand why this condition is achieved only at some spectral region we must analyse the parameter $\xi_1 = q_{1z}k_0d_1$. Because k_0 and d_1 are just positive constants, we will focus on the q_{1z} term which can be written as follows:

$$q_{1z} = q\sqrt{-\frac{1}{\varepsilon_{1z}}\left(\varepsilon_{1x}\cos^2\varphi + \varepsilon_{1y}\sin^2\varphi + 2\varepsilon_{1xy}\cos\varphi\sin\varphi\right)} = q\sqrt{-\frac{\varepsilon_{MoO_3}^{\varphi}}{\varepsilon_{1z}}}$$

Let us assume that the permittivities in the α-MoO_3 layer are purely real. This assumption is reasonable when there are small losses[3], i.e. when $\mathrm{Re}(\varepsilon_j) \gg \mathrm{Im}(\varepsilon_j)$ for $j = x, y, xy, z$. Again, we can assume that $\mathrm{Im}(q) = 0$ and $\mathrm{Re}(q) \gg \mathrm{Im}(q)$ as it is typically reported in symmetric bilayer stacks of α-MoO_3[4-7]. Under these assumptions, the term q_{1z} is either purely real or imaginary. Because $\varepsilon_{1z} > 0$ in this spectral regime, it will depend on the sign of the in-plane projected permittivity, $\varepsilon_{MoO_3}^{\varphi}$. If $\varepsilon_{MoO_3}^{\varphi} > 0$, then q_{1z} is purely imaginary and consequently, sufficiently large values of k allow to fulfil the condition $\tan(\xi_1) = i$. However, if $\varepsilon_{MoO_3}^{\varphi} < 0$, then q_{1z} is purely real which means that ξ_1 will not have an imaginary part that allows to fulfil $\tan(\xi_1) = i$. This is consistent with the analysis made in Fig. 3 of the main manuscript, where the good fit between the IFCs of the symmetric bilayer and the twisted asymmetric homostructure emerges at the region where the projected permittivity of the bottom layer is positive.

A similar study can be carried out for the thickness of the top layer. Interestingly, in this case the condition $\tan\xi_2 = i$ is satisfied for the complementary asymptotic region of the symmetric bilayer IFC (Supplementary Fig. 4d). Increasing the thickness of the top α-MoO_3 layer does not affect the IFC at this region for high momentum values.

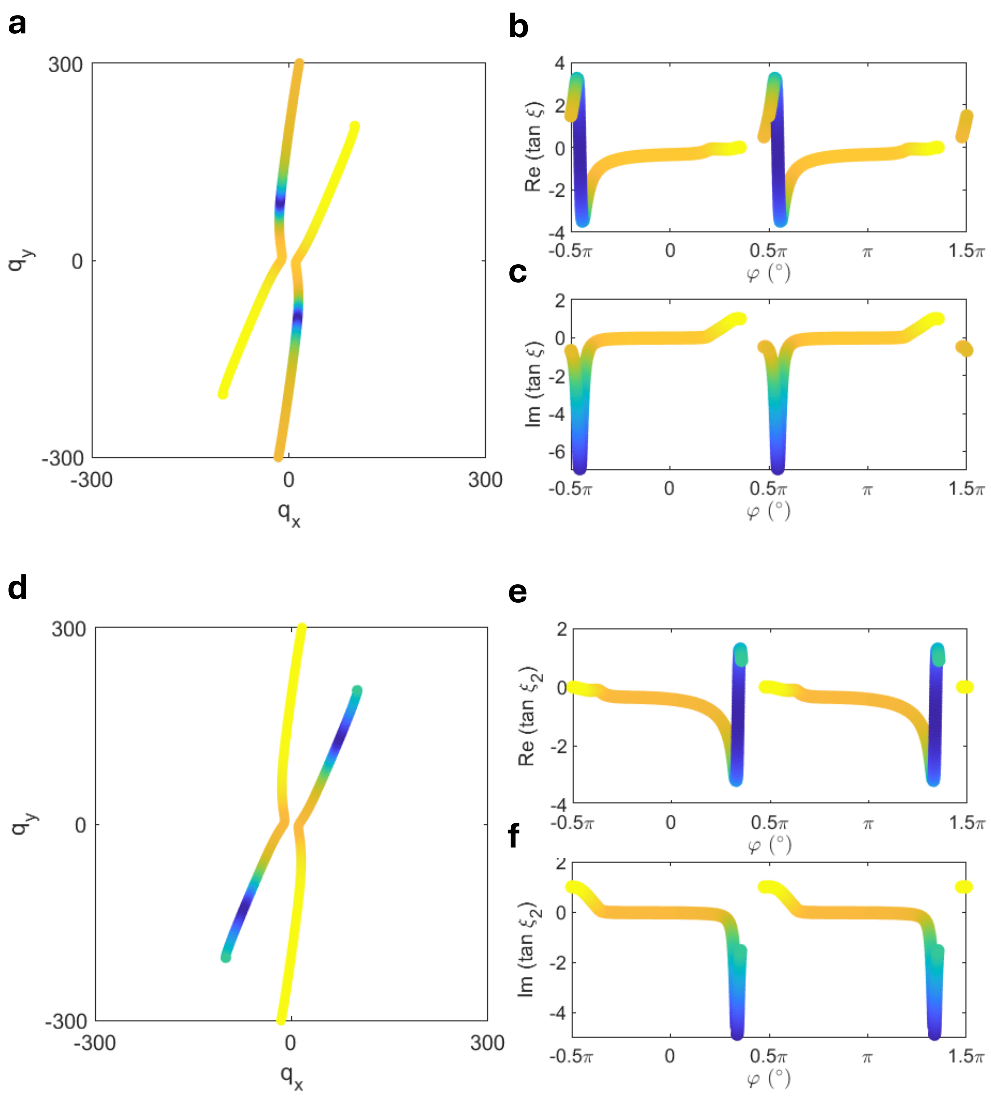

Supplementary Figure 4: Analytical study of the condition $\tan \xi = i$ in the polaritonic IFC of a twisted symmetric bilayer. a,d. Analytical IFC of a twisted ($\theta = 30^{o}$) symmetric bilayer made of two 80 nm-thin α-MoO₃ layers at an illumination frequency $\omega = 900\ \mathrm{cm}^{-1}$. **b,c.** Real (**b**) and imaginary part (**c**) of the term $\tan(\xi_1)$ as a function of the in-plane angle φ. **e,f.** Real (**e**) and imaginary part (**f**) of the term $\tan(\xi_2)$. The colorplots correspond to the value $\mathrm{Im}(\tan(\xi_i))$ for the bottom layer $i = 1$ (**a-c**) and the top layer $i = 2$ (**d-f**).

Supplementary Note 5. Analysis of the polaritonic propagation in a twisted ($\theta = 60^{o}$) homostructure as a function of thickness disparity

In this section we explain the appearance of the pinwheel polaritonic pattern at an homostructure stack with a twisted angle $\theta = 60^{o}$. As in Fig. 3 of the main manuscript, we study the polaritonic transition (with respect to the polaritonic propagation) from a symmetric α-MoO₃ twisted bilayer to an asymmetric twisted homostructure. In particular, we consider a bilayer with a twist angle $\theta = 60^{o}$ illuminated at $\omega = 900\ \mathrm{cm}^{-1}$ in which the thickness of the bottom layer varies from $d_{bot} = 80\ \mathrm{nm}$, to $500\ \mathrm{nm}$ and $3\ \mu\mathrm{m}$ while the thickness of the top α-MoO₃ layer is kept constant to $d_{top} = 80\ \mathrm{nm}$ (see schematics in Supplementary Fig. 6a-c). For

the twist angle considered, the symmetric bilayer (Supplementary Fig. 5a) exhibits canalization of PhPs (Supplementary Fig. 5e), yielding two flat bands in the momentum-space representation (Supplementary Fig. 5i). By increasing the bottom layer thickness to $500\ \mathrm{nm}$ (Supplementary Fig. 5b), we observe two distinct effects: (i) several parallel polaritonic fringes propagate along the same spatial direction of canalized PhPs in the symmetric twisted bilayer, and (ii) new polaritonic fringes, with almost horizontal wavefronts, appear propagating close to the vertical direction. Both effects maximize for the case of the thickest bottom layer (Supplementary Fig. 5c), in which the two patterns mix generating the pinwheel propagation (Supplementary Fig. 5g) observed in Fig. 2d,h of the main manuscript.

Analysing the FFT curves (Supplementary Fig. 5i-k), we realise that the shape of the IFCs for the asymmetric stacks tend to the IFC of the symmetric bilayer (white dashed curve in Supplementary Fig. 5i-k) for the thicker bottom layers. As in the case of a twisted stack with $\theta = 30^{\mathrm{o}}$, this effect occurs at the angular sector where the in-plane projected permittivity of the bottom layer exhibits positive values. This region corresponds to the narrow angular cone contained between the white and orange lines in Supplementary Fig. 5i-l, corresponding to the asymptotes of the IFC for the bottom and top α-MoO_3 layers considered alone, respectively. It is noteworthy that the confinement of the polaritonic modes decreases with the bottom layer thickness, which translates into IFCs with smaller momentum values close to the origin. When the bottom layer is sufficiently thick (Supplementary Fig. 5k), the polaritonic excitation in the stack is dominated by the weakly-confined polaritonic modes in the bottom layer. Considering the thick bottom layer alone (Supplementary Fig. 5d) we resemble the IFC of the stacked homostructure in this region of momentum space. Consequently, the physics behind the generation of the pinwheel polaritonic propagation is very similar to that of the 30°-twisted homostructure. One part of the IFC for the symmetric bilayer (exhibiting polariton canalization) couples with one of the asymptotes of the IFC for the single thick layer generating the pinwheel pattern. However, these two parts of the IFC are less aligned than in the 30°-twisted case. This results into an IFC with a significant curvature, responsible for the emergence of several curved fringes covering a narrow angular sector in real space.

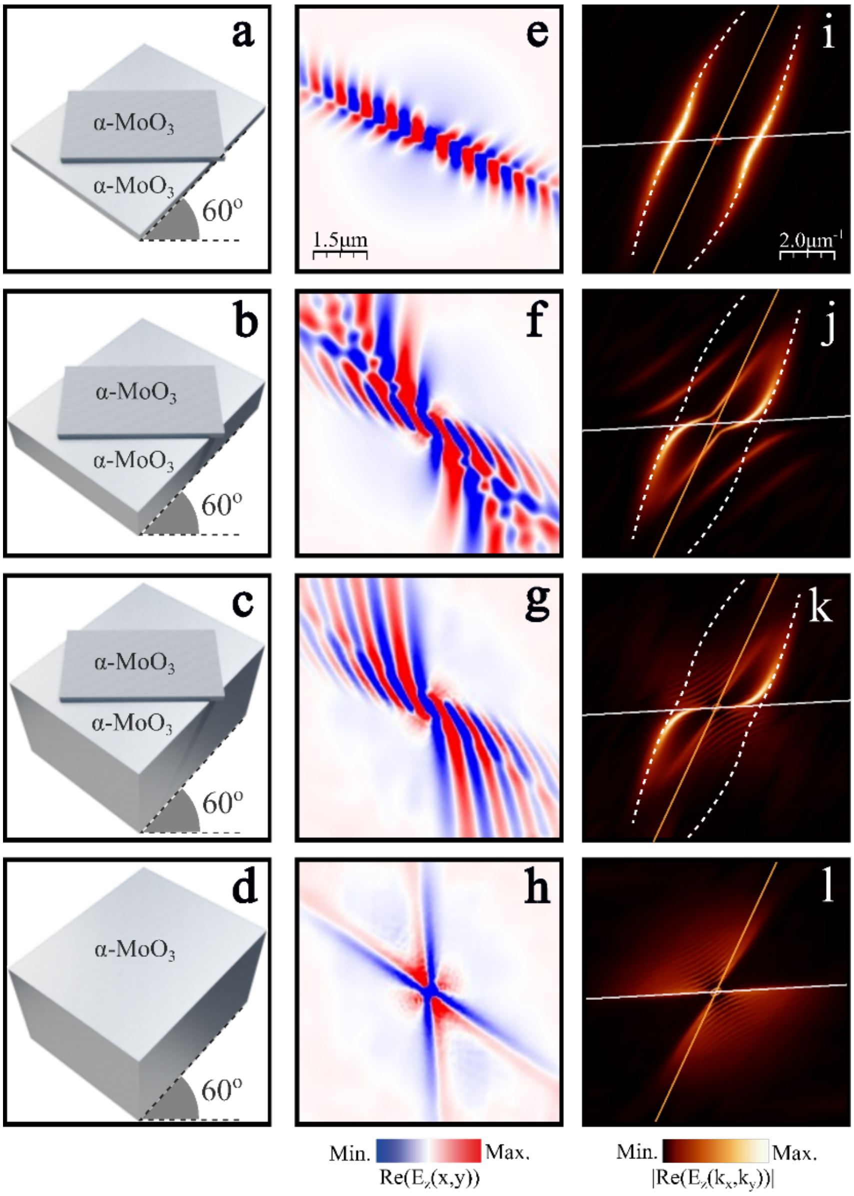

Supplementary Figure 5: Analysis of the polariton propagation as a function of thickness disparity in twisted homostructures. a-d. Schemes of the systems analysed. The top thin layer in **a-c** has a thickness $d_{top} = 80$ nm while the bottom layer has a thickness $d_{bot} = 80$ nm (**a**), $d_{bot} = 500$ nm (**b**), and $d_{bot} = 3$ µm (**c**). **d** corresponds to a single layer of α-MoO_3 with thickness $d = 3$ µm. In all cases, the twist angle is $\theta = 60^{\circ}$ and the illumination frequency is $\omega = 900\ \mathrm{cm}^{-1}$. **e-h.** Numerical simulations showing the electric field component $\mathrm{Re}(E_z)$ generated by a point dipole located above the four structures represented in **a-d**, respectively. **i-l.** IFCs obtained by performing the 2D-FFTs of the near-field simulations in **e-h**, respectively. White-dashed curves in **i-k** correspond to the analytic IFC of the bilayer case shown in **a**. White and orange lines in **i-l** are the asymptotes of the IFCs of PhPs in the bottom and top layers, respectively.

Supplementary Note 6. Analysis of the URPs response as a function of twist angle and illumination frequency in the homostructure stack

In this section we study the fundamental properties of the URPs arising at the homostructure. To do so, we extract from numerical simulations the direction of the rays, their intensity, and propagation lengths for several combinations of relative twist angles and illumination frequencies (Supplementary Fig. 6). Two different strategies have been followed to extract these

values. Let us consider one example: in Supplementary Fig. 6a,c we show numerical calculations of the absolute value of the polaritonic electric field $|E_z|$ at the top interface of the homostructure for a twist angle of $\theta = 30^{\text{o}}$ and an illumination frequency of $\omega = 920\ \text{cm}^{-1}$. To obtain the direction of propagation of the rays we extract a circular cut profile of radius $2\ \mu\text{m}$ centered at the in-plane coordinates of the dipole source (brown circle in Supplementary Fig. 6a and blue points in Supplementary Fig. 6b). This chosen radius is enough to properly observe the different behavior between the two rays. The results show two differentiated peaks at a small angular range, which correspond to the two rays observed in the simulated image (Supplementary Fig. 6a). Remarkably, there is a significant difference between the maximum intensity of the two peaks, corresponding to the enhanced and suppressed ray. To extract the direction of these rays as well as the maximum intensity, we fit this profile to a linear combination of two gaussian functions:

$$y(x) = a_1 exp\left(-\left(\frac{x - b_1}{c_1}\right)^2\right) + a_2 exp\left(-\left(\frac{x - b_2}{c_2}\right)^2\right) + d$$

The fitting curve (brown continuous curve in Supplementary Fig. 6b) shows a good agreement with the extracted profile. Note that the calculated coefficients, a_i, b_i corresponds to the maximum intensity and direction of propagation of the enhanced ($i = 1$) and suppressed ($i = 2$) ray, respectively. Repeating the process for two different twist angles, $\theta = 0^{\text{o}}$ and 15°, we extract the remaining profiles (blue and purple curves in Supplementary Fig. 6b). Two symmetric rays, with similar intensity, arise at $\theta = 0^{\text{o}}$. Thus, we observe that increasing the twist angle enhances the intensity of one ray and reduces the intensity of the other, generating the observed URP propagation. A similar procedure is followed to obtain the propagation lengths of the rays. In particular, we extract linear profiles along the enhanced or suppressed ray directions (1 and 2 in Supplementary Fig. 6c, respectively), which were taken from the previous profile analysis. The starting point of the profile is set at $1\ \mu\text{m}$ far from the dipole source. Thus, the extracted field profile is mostly generated by the URP field but with a small contribution from the dipole source. We observe a non-oscillatory decay for the three twist angles consistent with the presence of a constant phase ray. By fitting these curves to an exponential decay:

$$y(x) = a\ exp(-bx) + c$$

we can extract the propagation length of the rays, defined as the distance at which the initial field within a ray is reduced by a factor of 1/e, i.e as $L_p = \frac{1}{b}$. A summary of these properties for the twist angles $\theta = 0^{\text{o}}$, 15° and 30° and illumination frequencies $\omega = 880\ \text{cm}^{-1}$, $900\ \text{cm}^{-1}$ and $920\ \text{cm}^{-1}$ is displayed in Supplementary Fig. 6e-g. Continuous lines correspond to the enhanced ray values while dashed lines represent those that are suppressed. Several conclusions can be extracted from these figures. First, the direction of propagation of the suppressed ray evolves linearly with the twist angle. As described in the main manuscript, this ray comes from the single thick layer system, so it is almost independent of the relative twist angle between the layers. Moreover, the intensity barely changes with the twist angle. There is only a small reduction of the polaritonic field due to the change of the in-plane permittivity of the thin top α-MoO_3 layer while twisting. On the other hand, the direction of propagation of the enhaced ray follows a similar behaviour but with less variation. As shown in sections S2 and S3, increasing the twist angle as well as reducing the illumination frequency values enhances the number of fringes that constitute the URPs. This feature, represented in the momentum space by an increasement of the slope of the URP IFC is responsible for the imperfect linear behavior. Remarkably, the maximum intensity values of the enhaced rays strongly increase with the twist angle. The most prominent case corresponds to an illumination frequency of $\omega = 880\ \text{cm}^{-1}$ where this value increases by a factor of 5 for $\theta = 30^{\text{o}}$. Surprisingly, the propagation lengths of the enhaced and suppressed rays barely change with the relative twist angle and illumination frequency. Thus, losses do not play a major role at this system and the URP generation is purely

an amplitude effect. This suggests that the unidirectionality effectively emerges from a modulation of the effective mode density for each ray in the homostructure.

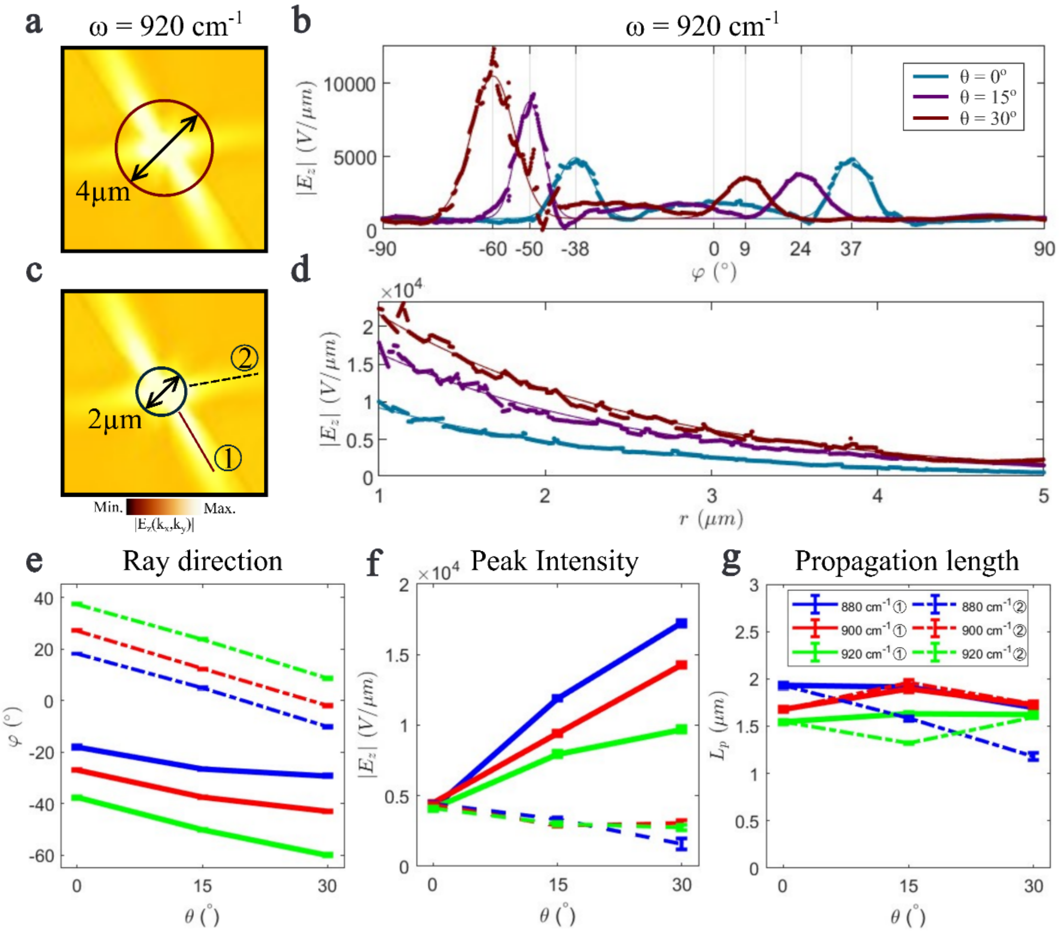

Supplementary Figure 6: Analysis of the URP propagation as a function of relative twist angle and illumination frequency in the homostructure stack. a,c. Numerical simulations showing the absolute value of the electric field component $|E_z|$ generated by a point dipole in an homostructure stack with a twist angle of $\theta = 30^{\circ}$ and an illumination frequency $\omega = 920\ \mathrm{cm}^{-1}$. **b,d.** Profiles of the numerical simulations along circular cuts at 2 μm far from the dipole source (brown circle in **a**) and linear cuts along the direction of propagation of the enhanced ray (continuos brown line in **c**). The illumination frequency is fixed to $\omega = 920\ \mathrm{cm}^{-1}$ while the twist angle varies as $\theta = 0^{\circ}$, 15°, and 30° (blue, purple and grown curve, respectively). The extracted profiles are fitted to a linear combination of two gaussian functions for **b** and to an exponential function for **d**. **e-g.** Ray direction, maximum peak intensity, and propagation lengths of the enhanced (continuous curves) and suppressed rays (dashed curves) as a function of twist angle ($\theta = 0^{\circ}$, 15° and 30°) and illumination frequency ($\omega = 880\ \mathrm{cm}^{-1}$, $900\ \mathrm{cm}^{-1}$ and $920\ \mathrm{cm}^{-1}$).

Supplementary Note 7. Determining the twist angle between a-axes of monoclinic β-Ga_2O_3 and α-MoO_3 using polarization-resolved Raman spectroscopy

A beta-phased gallium oxide (β-Ga_2O_3/bGO) unit cell comprises 10 atoms (4 Ga-atoms and 6 O-atoms) which contribute 3 acoustic and 27 optical phonon branches to the phonon dispersion curve within the first Brillouin zone[8,9]. An irreducible representation of these phonon modes at Γ-point can be written as:

$$\Gamma = 10A_g + 5B_g + 5A_u + 10B_u$$

Where $A_u + 2B_u$ and $10A_g + 5B_g + 4A_u + 8B_u$ correspond to the acoustic and optical phonons, respectively. Among optical phonons, the modes with Au and Bu symmetry have odd parity and are infrared active (Raman inactive), whereas modes with Ag and Bg symmetry have even parity (Raman active) and can be probed through first-order Raman scattering.

To determine the crystallographic direction of β-Ga_2O_3, we performed polarization-resolved Raman spectroscopy on the (010) plane, which represents the monoclinic plane of β-Ga_2O_3. The selection rule predicts the presence of Ag modes and the absence of Bg modes while excitation is parallel polarized to the (010) plane. We performed polarization-resolved Raman spectroscopy using WiTec alpha 300R Raman spectrometer in the backscattering geometry in which the polarization state of incident light is kept parallel to the analyzer. We rotate the polarization state of incident light from -90° to 90° in the step of 10°. We used a 532 laser line as an excitation source and illuminated (and collected) the surface using a 5x/0.13NA objective. Raman scattered light was dispersed through a grating of 300 lines/mm.

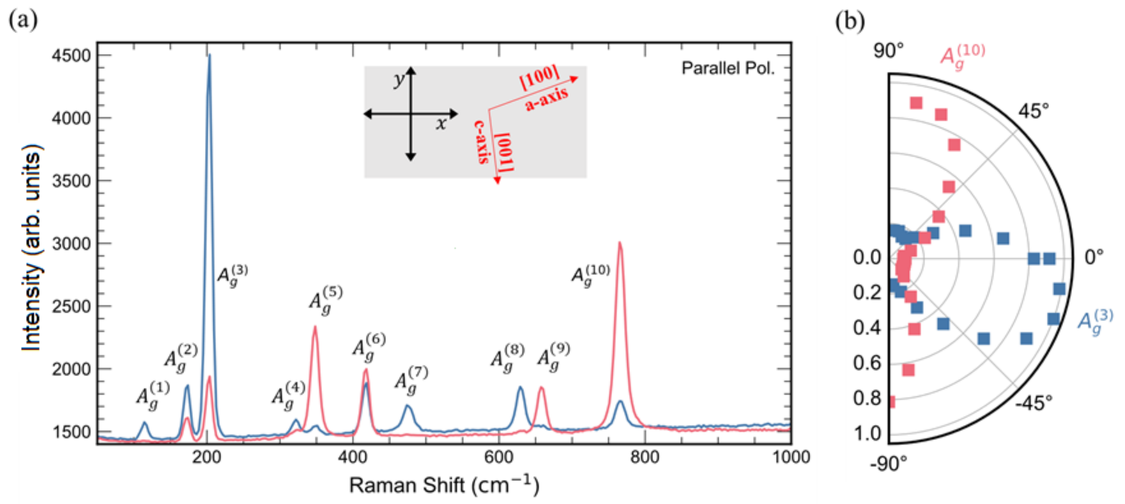

Supplementary Fig. 7. Polarization Resolved Raman Spectroscopy of β-Ga_2O_3. a. Raman spectra with different first-order Raman active modes of β-Ga_2O_3 obtained in the parallel polarization configuration for incident light parallel to x (blue) and y (red) axis. **b.** Variation of intensity of Ag3 and Ag10 modes as a function of the orientation of the polarization state of incident light in XY plane.

In the Porto notation, we can write the scattering geometry as (i) z(xx)z' and (ii) z(yy)z' where x, y, and z correspond to standard lab coordinate frames (z' is the back-scattering direction). Supplementary Fig. 7a shows polarization-resolved Raman spectra of the β-Ga_2O_3 substrate, where the red and blue color spectrum represents polarization parallel to the x-axis and y-axis, respectively. The first-order Raman modes Ag1, Ag2, Ag3, Ag4, and Ag7 exhibit maximum intensity in z(xx)z' geometry, whereas Ag5, Ag6, Ag9, and Ag10 exhibit maximum intensity in z(yy)z', indicating 90 degrees phase shift in two sets of Raman modes. Hence, we plot Ag3 and Ag10 in the polar plot, as shown in Supplementary Fig. 7b, as a function of the azimuthal rotation angle of the polarization state of incident light. We find the maximum intensity of Ag3 is 20 degrees off in the counter-clockwise direction from the x-axis in the lab coordinate frame, which corresponds to the a-axis of β-Ga_2O_3. After finding the a-axis of β-Ga_2O_3, we next calculate the angle between a-axes of β-Ga_2O_3 and α-MoO_3 (which is represented by the short side of the α-MoO_3 layer) and tabulated below. In the Supplementary Table 1, the negative and positive

angles represent counter-clockwise and clockwise directions of mis-orientation of a-axes of β-Ga_2O_3 w.r.t. α-MoO_3 layers. With regard to the twist angles shown in the main text we have added a 20-degree deviation to adjust the instrumental least count while increasing the alignment with our computational results.

Flake No	Thickness (nm)	Twist (Degrees)
1	220	65
2	400	20
3	198	120

Supplementary Table 1. Thicknesses of α-MoO_3 flakes under investigation and orientation of their [100] crystallographic orientation w.r.t. bGO [100] orientation.

Supplementary Note 8. Near-field s-SNOM visualization of PhPs propagating in asymmetric heterostructures

Fig. 4 in the main text showed s-SNOM imaging of PhPs in thin α-MoO_3 layers deposited on β-Ga_2O_3 for a frequency $\omega = 734\,\mathrm{cm}^{-1}$ and twist angles $\theta = 45^{o}$, 0° and 100°. Here, we additionally display s-SNOM images of the same samples for the additional frequencies $\omega = 720\,\mathrm{cm}^{-1}$ and $\omega = 750\,\mathrm{cm}^{-1}$ (see Supplementary Fig. 8a). We also show the corresponding full-wave simulations for the same material structure (see Supplementary Fig. 8b). While we find the highest degree of anisotropy between the rays and, therefore, the highest directionality of the propagation, for the twist angles $\theta = 45^{o}$ and 100° at $\omega = 734\,\mathrm{cm}^{-1}$, high ray-anisotropy can also be found for $\theta = 100^{o}$ at $\omega = 750\,\mathrm{cm}^{-1}$ and for $\theta = 45^{o}$ at $\omega = 720\,\mathrm{cm}^{-1}$ and $\omega = 750\,\mathrm{cm}^{-1}$. These results demosntrate that unidirectional ray propagation is not limited to only one specific twist-angle frequency pair. Notably, the change of anisotropy is only minimal at the measured frequencies for a twist angle of $\theta = 0^{o}$.

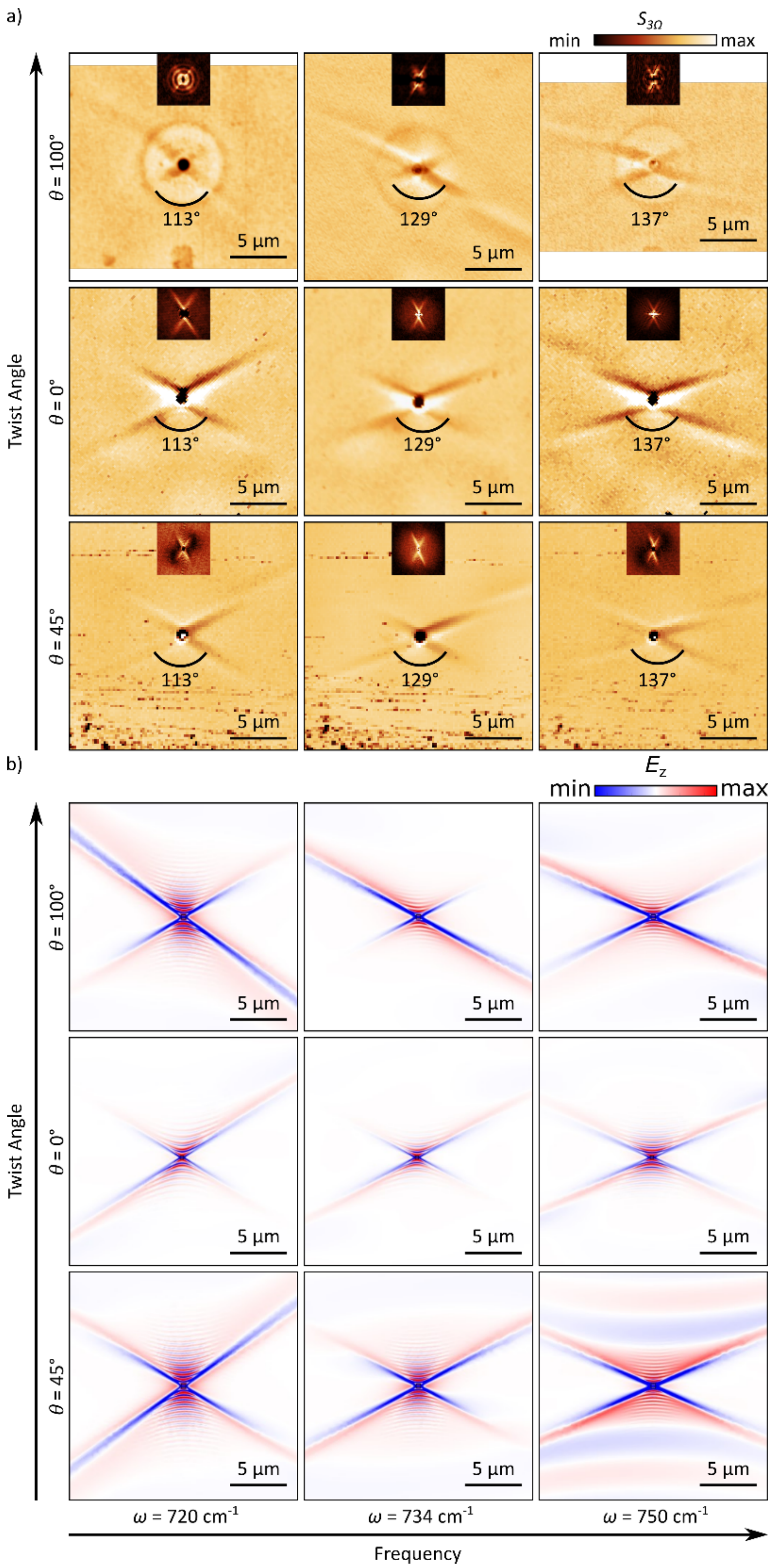

Supplementary Figure 8: Measured frequency dependence of the ray-like polariton propagation in thin α-MoO_3 layers placed on β-Ga_2O_3. a. s-SNOM images taken by using a FEL. The measurements show a ray-like polariton propagation with a noticeable anisotropy between the two rays for the twist angles $\theta = 45^o$ and 100°, while no specific anisotropy is found for $\theta = 0^o$. **b.** Full-wave simulations performed using the same sample-parameters as in **a** mirror the experimental results, confirming the good match between theory and experiment. The simulations predict a slight anisotropy for all twist-angle frequency pairs, but a notable increased anisotropy for the twist angles $\theta = 45^o$ and 100°.

Supplementary Note 9. Explanation of the ray appearance as a function of the substrate permittivity values

To understand the intriguing ray-like propagation observed, we perform full-wave numerical simulations of a simplified sample-system with a 100 nm thick slab of α-MoO_3 deposited on an artificial isotropic substrate with permittivities between +2 and -4 (see Supplementary Fig. 9). We find that a decrease of the substrate permittivity leads to a decrease of the momentum values in the hyperbolic IFCs of the polaritons propagating in the α-MoO_3 layer. In this process, the basis of the hyperbolas shortens until it vanishes, resulting in the vanishing of the corresponding polaritonic fringes and the emergence of the ray-like propagation. Notably, the emergence of rays is a continuous process and does not require the hyperbolic IFCs to touch each other. While the optimal case arises for a substrate permittivity of -1, close negative values like -0.5 and -2 also exhibit ray-like PhPs propagation. We conclude that the observed ray-like propagation in the α-MoO_3/β-Ga_2O_3 heterostructures is not a feature of the anisotropy of β-Ga_2O_3, but of the low negative values of the permittivity in the measured frequency range. Remarkable, high negative permittivity values $\varepsilon_{substrate} = -4$ results into a flip of the propagation direction. In-plane hyperbolic PhPs propagate within an angular sector centered along the x axis (the [100] α-MoO_3 axis) which corresponds to a previous forbidden direction in this frequency range. A similar effect has been reported in ref. 10.

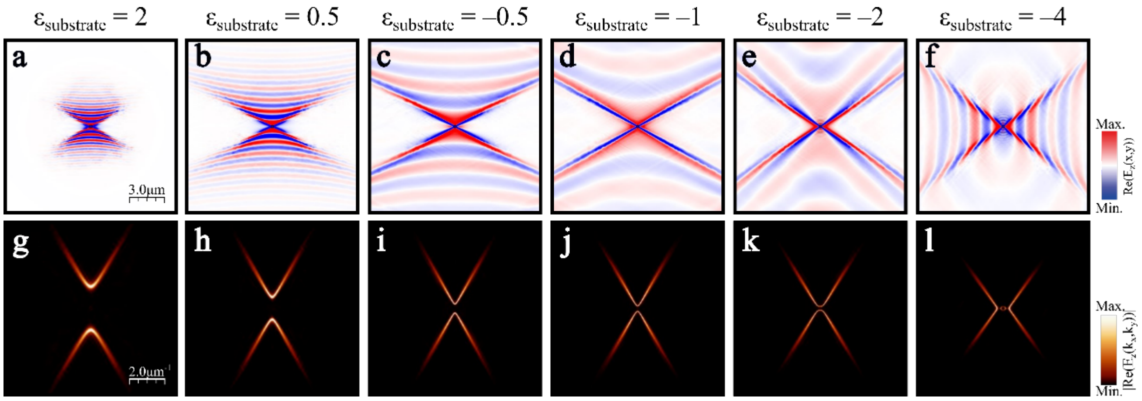

Supplementary Figure 9: Substrate-permittivity dependence of ray-like propagation. a-f. Simulated near-field amplitude images of a system consisting of a 100 nm-thick α-MoO_3 layer placed on an artificial isotropic substrate with permittivity $\varepsilon_{substrate} = 2, 0.5, -0.5, -2$ and -4 (from left to right) calculated by full-wave numerical simulations. **g-l.** Simulated IFCs of **a-f** obtained by performing the 2D-FFTs of the simulated near-field images **a-f**, respectively. As the substrate permittivity decreases, the hyperbolic IFCs move towards each other and lose their basis, corresponding to a decrease of hyperbolic fringes.

Supplementary Note 10. Impact of an anisotropic substrate on the polariton propagation

Due to the optical anisotropy of β-Ga_2O_3, a study of the impact of a substrate with anisotropic permittivity on the polariton propagation is necessary. To this end, we utilize the formula in Equation (S4) and derived in S1 to calculate the real and imaginary part of the momentum of a polariton ($\mathrm{Re(k)}$ and $\mathrm{Im(k)}$) propagating in an artificial material with an out-of-plane permittivity $\varepsilon_z = +5$ and an in-plane permittivity $\varepsilon_\varphi = +5$ (Supplementary Fig. 9a,c) and -5 (Supplementary Fig. 9b,d). The material is located on an artificial uniaxial substrate whose permittivities in the in-plane direction $\varepsilon_{\varphi,substrate}$ and out-of-plane direction $\varepsilon_{z,substrate}$ are individually varied between -4 and $+4$ (see Supplementary Fig. 9). The superstrate permittivity is set as $\varepsilon_{superestrate} = 1$. Note that all permittivities are assumed to have a negligible imaginary part of 0.001.

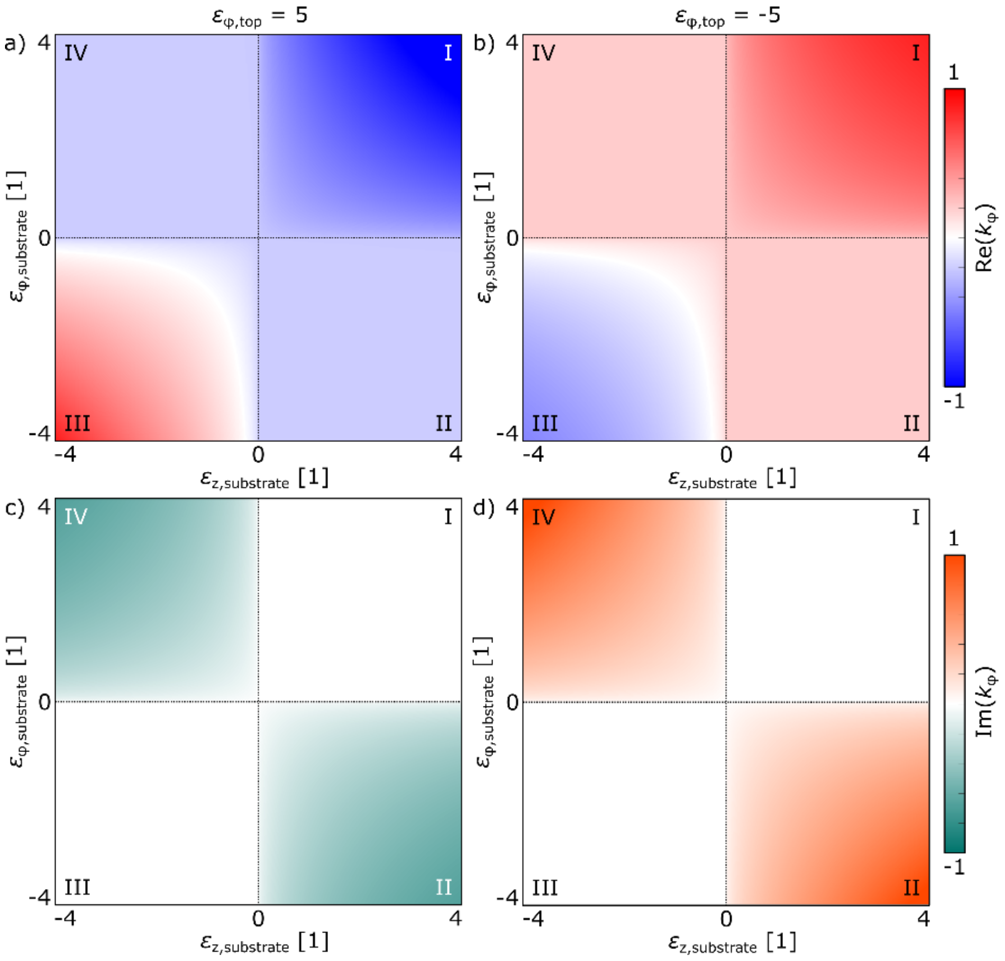

Supplementary Figure 10: Real and imaginary part of the polariton momentum propagating in the direction φ of an anisotropic substrate. The permittivity of the superstrate is set to $\varepsilon_{superestrate} = 1$, the permittivity of the top layer in the out-of-plane direction is set to $\varepsilon_z = 5$ and in the in-plane direction to $\varepsilon_\varphi = 5$ (**a,c**) and -5 (**b,d**). The polariton momentum is plotted as a function of the substrate permittivity in the out-of-plane direction ($\varepsilon_{z,substrate}$) along the x-axis and in the in-plane direction ($\varepsilon_{\varphi,substrate}$) along the y-axis.

Highly confined PhPs in a finite layer placed over a positive substrate are expected to exist along the layer direction with a negative permittivity (quadrant I in Supplementary Fig. 10b). Opposite signs of the in-plane and out-of-plane substrate permittivity results into less confined and highly

damped PhPs (quadrant II and IV in Supplementary Fig. 10b). No polariton propagation is expected when the substrate permittivity is completely negative (quadrant III in Supplementary Fig. 10b). In contrast, positive permittivity values of the layer (Supplementary Fig. 10a) translate into negative momentum values $\mathrm{Re(k)} < 0$, which can be understood as in-plane forbidden directions for polaritons. Polariton propagation only appears for high negative values of the substrate permittivity (quadrant III in Supplementary Fig. 10a). This is consistent with the flipped propagation observed in S9 for an isotropic substrate with permittivity $\varepsilon_{substrate} = -4$. Remarkably, a reduction of Re(k) towards zero (white region in Supplementary Fig. 9a,b), and therefore a ray-like propagation, can only be expected when both the in-plane and out-of-plane permittivities are negative. This white region in quadrant III of Supplementary Fig. 10 a and b symbolizes Re(k) = 0, corresponding to perfect ray-like propagation. It represents the analogous -1 permittivity condition but generalized for an anisotropic substrate. It can be calculated by the following formula derived from Equation (S4):

$$\varepsilon_{z,superstrate} + \varepsilon_{z,substrate}\sqrt{\frac{\varepsilon_{x,substrate}}{\varepsilon_{z,substrate}}\cos^2\varphi + \frac{\varepsilon_{y,substrate}}{\varepsilon_{z,substrate}}\sin^2\varphi} = 0$$

Assuming that the substrate permittivity values involve are purely real and negative, we can simplify this expression as follows:

$$\varepsilon_{z,superstrate} - \sqrt{\varepsilon_{z,substrate}\varepsilon_{\varphi,substrate}} = 0$$

The -1 permittivity condition can be easily recovered by considering air as a superstrate and an isotropic substrate.

Supplementary Note 11. Evolution of the β-Ga_2O_3 permittivity in the explored frequency region

In the frequency-range from 705 to $765\mathrm{cm}^{-1}$, the permittivity of β-Ga_2O_3 presents almost always negative values in the zz-direction (data taken from ref. 11): $\varepsilon_{zz} = -3.04 + 0.11i$ for $\omega = 705\ \mathrm{cm}^{-1}$ and $\varepsilon_{zz} = 0.11 + 0.005i$ for $\omega = 765\ \mathrm{cm}^{-1}$. Likewise, the adjusted in-plane direction nn also shows negative permittivities between $\varepsilon_{nn} = -8.23 + 0.48i$ for $\omega = 705\ \mathrm{cm}^{-1}$ and $\varepsilon_{nn} = 0.22 + 0.05i$ for $\omega = 765\ \mathrm{cm}^{-1}$. The adjusted in-plane direction mm features positive permittivity-values from $\omega = 705\ \mathrm{cm}^{-1}$ to $\omega = 744\ \mathrm{cm}^{-1}$, at which point the permittivity switches to negative values. The angle of propagation in β-Ga_2O_3 [11], defined by Equation (S5), changes in this frequency range from $\gamma = -2.7^{\mathrm{o}}$ to -43.8^{o}.

$$\gamma(\omega) = \frac{1}{2}\arctan\left(\frac{2\mathrm{Re}(\varepsilon_{xy})(\omega)}{\mathrm{Re}(\varepsilon_{xx})(\omega)-\mathrm{Re}(\varepsilon_{yy})(\omega)}\right) \qquad \text{Eq. S5}$$

As described in sections S9 and S10, the ray-like shape of the propagation emerges at negative permittivities of the substrate in the in-plane and out-of-plane directions and culminates when the hyperbolic IFCs intersect each other at around a permittivity value of -2, while polaritonic fringes appear again if the substrate permittivity has higher values. Therefore, the increase of the permittivity of β-Ga_2O_3 in nn- and zz-direction for higher frequencies explains the re-emergence of the fringes, as seen in Supplementary Fig. 8b (section S8). Additionally, we see that β-Ga_2O_3 features an in-plane anisotropic damping, contributing to the observed ray anisotropy. Furthermore, in the frequency range between $\omega = 705\ \mathrm{cm}^{-1}$ and $\sim 743\ \mathrm{cm}^{-1}$, β-

Ga_2O_3 features one in-plane direction with positive permittivity, while the permittivity in the out-of-plane direction is negative. As described in section S10, this also results in an increased damping along this direction and, therefore, an anisotropy between the propagating rays.

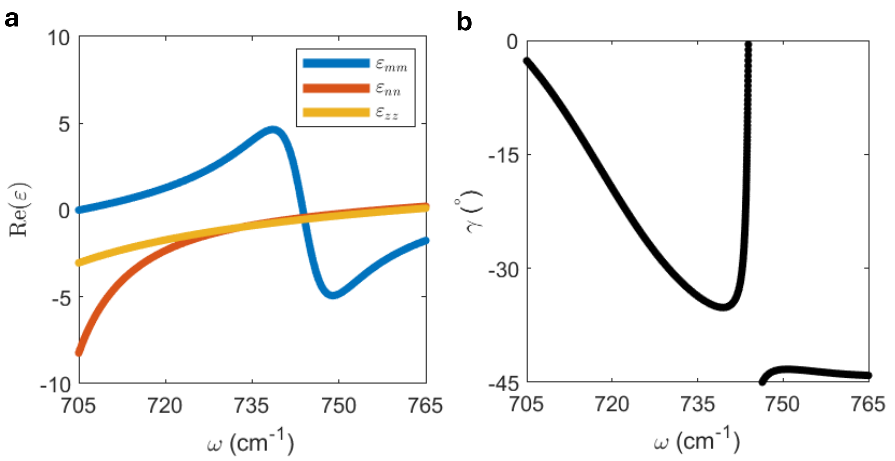

Supplementary Figure 11: Dielectric permittivity tensor of β-Ga_2O_3. a. Diagonal elements ε_{mm}, ε_{nn} and ε_{zz} of the frequency-dispersive β-Ga_2O_3 permittivity. **b.** Rotation angle γ (Equation (S5)) as a function of frequency.

Supplementary Note 12. Influence of the β-Ga_2O_3 permittivity on the ray-like propagation in α-MoO_3

In this section we show numerically the potential degree of control over the ray-like polariton propagation in the α-MoO_3/β-Ga_2O_3 heterostructure stack.

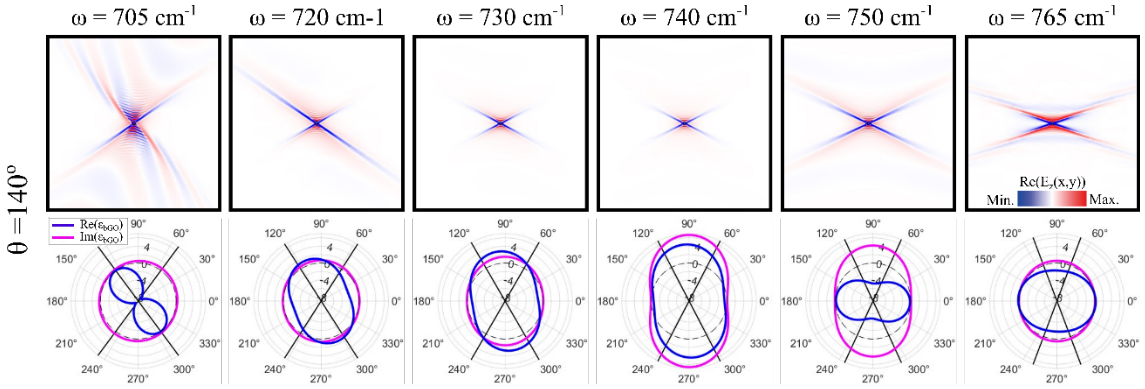

Supplementary Figure 12: Frequency-dependence of the ray-anisotropy for a constant twist angle $\theta = 140^o$ as calculated by full-wave simulations. The images in the top row show the simulated polariton propagation in real-space, while the graphs in the bottom show the real (blue line) and imaginary part (violet line) of the effective in-plane permittivity of the β-Ga_2O_3 substrate over the angle of propagation. The black lines hereby correspond to the direction of the asymptotes of the hyperbolic IFCs of the α-MoO_3 top layer. Due to the shear-effect, the effective β-Ga_2O_3 in-plane permittivity rotates over the frequency, therefore, enabling both symmetric and asymmetric interactions with the polariton propagating in the top-layer. This results in an unidirectional ray-like propagation for $\omega = 720\ \mathrm{cm}^{-1}$ and mostly symmetric rays for all higher frequencies. This shows that even with a mere change of frequency, tuning of the ray-like polariton propagation can be achieved.

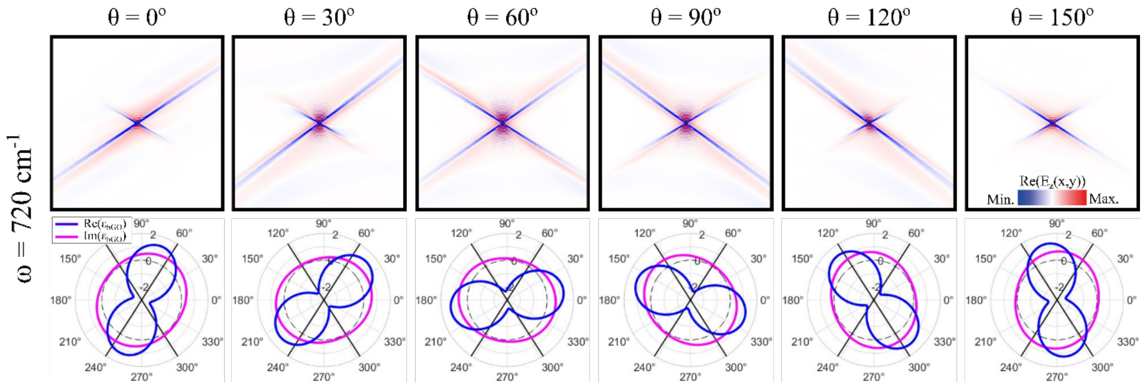

Supplementary Figure 13: Twist angle-dependence of the ray-anisotropy for a constant frequency $\omega = 720\ \mathrm{cm}^{-1}$ as calculated by full-wave simulations. The images in the top row show the simulated polariton propagation in real-space, while the graphs in the bottom row show the real (blue line) and imaginary part (violet line) of the effective in-plane permittivity of the β-Ga_2O_3 substrate over the angle of propagation. The black lines hereby correspond to the direction of the asymptotes of the hyperbolic IFCs of the α-MoO_3 top layer. Compared to the case shown in the main text for a frequency of $\omega = 734\ \mathrm{cm}^{-1}$, the angular range of negative effective in-plane permittivity of the β-Ga_2O_3 is larger and the additional damping due to an increased imaginary part of the in-plane permittivity is reduced, resulting in a lower range of twist angles showing low-propagative rays (seen here at $\theta = 150^{\mathrm{o}}$).

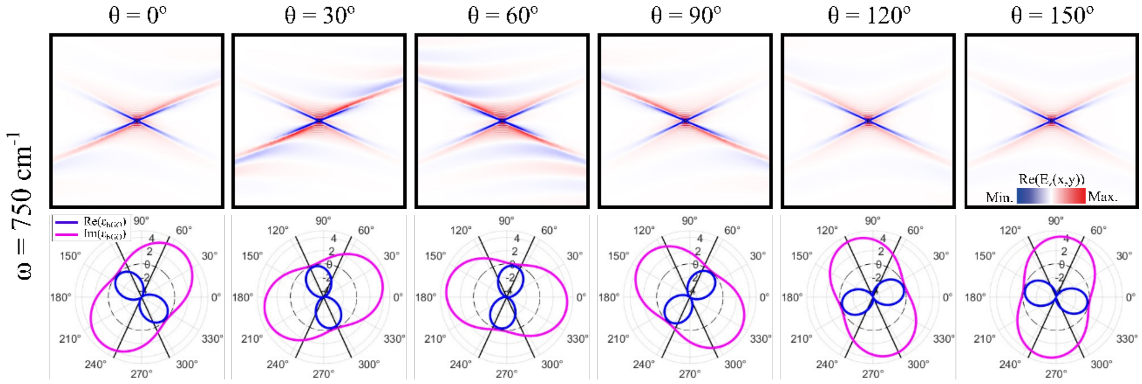

Supplementary Figure 14: Twist angle-dependence of the ray-anisotropy for a constant frequency $\omega = 750\ \mathrm{cm}^{-1}$ as calculated by full-wave simulations. The images in the top row show the simulated polariton propagation in real-space, while the graphs in the bottom row show the real (blue line) and imaginary part (violet line) of the effective in-plane permittivity of the β-Ga_2O_3 substrate over the angle of propagation. The black lines hereby correspond to the direction of the asymptotes of the hyperbolic IFCs of the α-MoO_3 top layer. As the effective in-plane permittivity of β-Ga_2O_3 is negative for all propagation angles φ, no additional damping is induced on the propagating polaritons due to opposite signs of in- and out-of-plane substrate permittivities. This leaves the anisotropic imaginary part of the effective in-plane permittivity of β-Ga_2O_3 as the only contribution to any ray anisotropy. As can be seen in the images, this contribution is not strong enough to yield unidirectional propagation of rays.

Supplementary Note 13. Analysis of the URPs response as a function of twist angle and illumination frequency in the heterostructure stack

Similarly to S6, we analyse the fundamental properties of URPs as a function of twist angle and illumination frequency in the heterostructure stack (Supplementary Fig. 15). First, as mentioned in the main manuscript, the direction of propagation of the rays remains constant independently of the twist angle and barely changes with frequency. Moreover, the intensity of the peaks dramatically changes with the twist angle. For instance, at $\omega = 720\ \mathrm{cm}^{-1}$ (Supplementary Fig. 15b) we observe URPs at each ray directions for $\theta = 0^{\mathrm{o}}$ and 120°, while a symmetric propagation is observed at $\theta = 60^{\mathrm{o}}$. Notably, a non-oscillatory field is revealed at the profiles along the direction of propagation. Like in the homostructure, gaussian and exponential decay fittings were made to extract the direction of propagation, the peak intensity of the rays and its propagation lengths. A summary of these properties is displayed in Supplementary Fig. 15e-g for several combinations of twist angles (from $\theta = 0^{\mathrm{o}}$ to 150° in 30° steps) and llumination frequencies ($\omega = 720\ \mathrm{cm}^{-1}$, $734\ \mathrm{cm}^{-1}$ and $750\ \mathrm{cm}^{-1}$). Remarkably, and in contrast to the homostructure, the intensity of the peak (Supplementary Fig. 15f) is strongly correlated with the propagation lengths (Supplementary Fig. 15g). Consequently, the URPs in this material system exhibit not only higher intensity values but also lower losses and larger propagation lengths than the symmetric cases. Overall, the response of this material system is different compared to the homostructure.

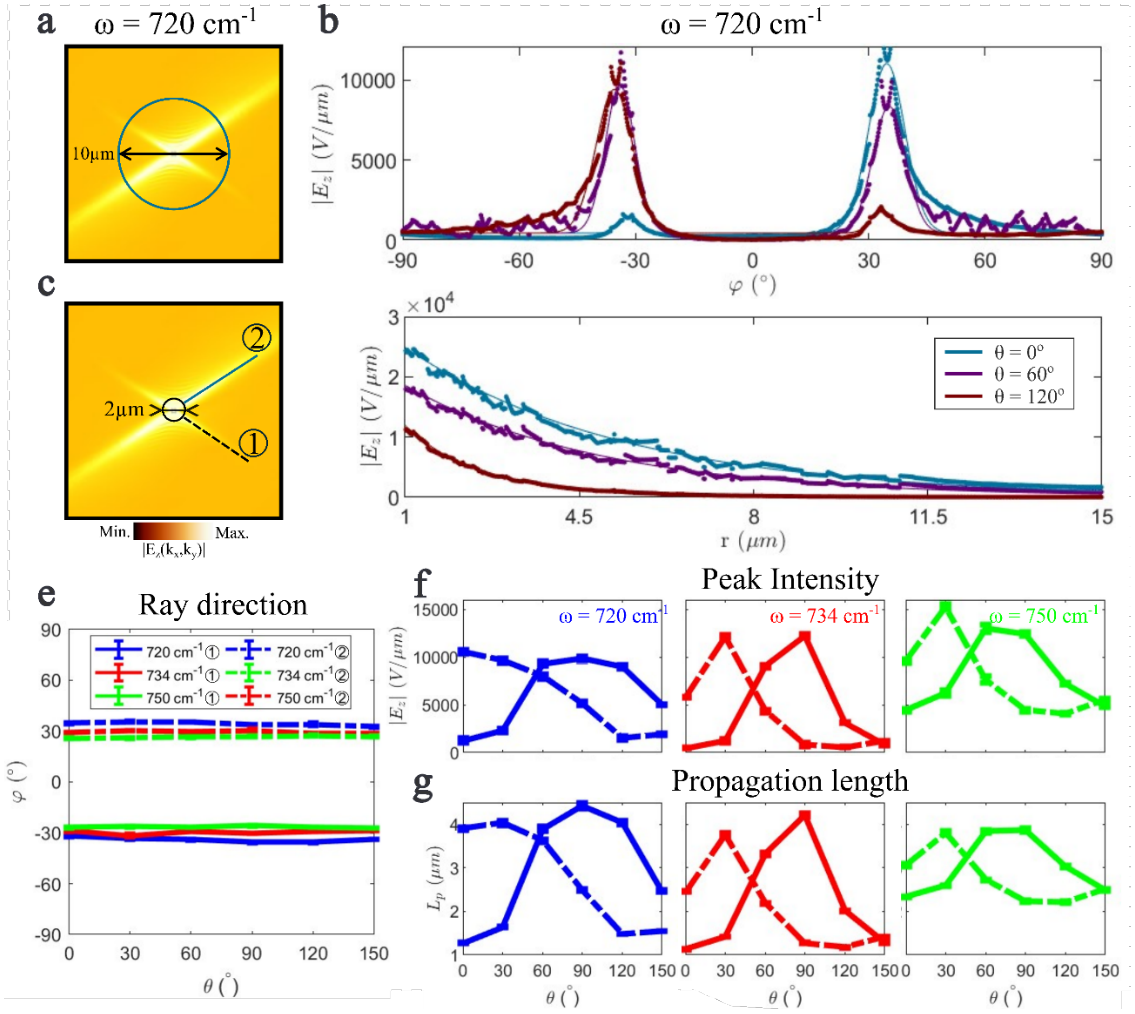

Supplementary Figure 15: Analysis of the URP propagation as a function of relative twist angle and illumination frequency in the heterostructure stack. a,c. Numerical simulations showing the absolute value of the electric field component $|E_z|$ generated by a point dipole in an heterostructure stack with a twist angle of $\theta = 0^o$ at an illumination frequency of $\omega = 720\ \mathrm{cm}^{-1}$. **b,d.** Profiles of the numerical simulations along circular cuts at 5 μm far from the dipole source (blue circle in **a**) and linear cuts along the direction of propagation of the enhaced ray (continuos blue line in **c**). The illumination frequency is fixed to $\omega = 720\ \mathrm{cm}^{-1}$ while the twist angle varies from $\theta = 0^o$, 60° to 120° (blue, purple and brown curve, respectively). The extracted profiles are fitted to a linear combination of two gaussian functions for **b** and to a exponential function for **d**. **e-g.** Ray direction, maximum peak intensity and propagation lengths of the two rays as a function of twist angle (from $\theta = 0^o$ to 150° in 30° steps) and illumination frequency ($\omega = 880\ \mathrm{cm}^{-1}$, $900\ \mathrm{cm}^{-1}$ and $920\ \mathrm{cm}^{-1}$). Continuous and dashed curves are consistently used for the same ray in figures **e-g**.

Supplementary References:

1. Duan, J., Álvarez-Pérez, G., Lanza, C. et al. Multiple and spectrally robust photonic magic angles in reconfigurable α-MoO3 trilayers. *Nat. Mater*. **22**, 867–872 (2023).
2. Álvarez-Pérez, G. et.al. Analytical approximations for the dispersion of electromagnetic modes in slabs of biaxial crystals. *Phys. Rev. B* **100**, 235408 (2019).
3. Álvarez-Pérez, G. et al. Infrared permittivity of the biaxial van der Waals semiconductor α-MoO3 from near-and far-field correlative studies. *Adv. Mater*. **32**, 1908176 (2020).

4. Hu, G., Ou, Q., Si, G. et al. Topological polaritons and photonic magic angles in twisted α-MoO_3 bilayers. *Nature* **582**, 209–213 (2020).
5. Chen, M., Lin, X., Dinh, T.H. et al. Configurable phonon polaritons in twisted α-MoO_3. *Nat. Mater*. **19**, 1307–1311 (2020).
6. Duan, J. et al. Twisted nano-optics: manipulating light at the nanoscale with twisted phonon polaritonic slabs. *Nano Lett*. **20**, 5323–5329 (2020).
7. Zheng, Z. et al. Phonon polaritons in twisted double-layers of hyperbolic van der Waals crystals. *Nano Lett.* **20**, 5301–5308 (2020).
8. Kranert, C.; Sturm, C.; Schmidt-Grund, R.; Grundmann, M. Raman Tensor Elements of β-Ga_2O_3. *Sci Rep* 2016, 6, 1–9.
9. Janzen, B. M.; Gillen, R.; Galazka, Z.; Maultzsch, J.; Wagner, M. R. First- and Second-Order Raman Spectroscopy of Monoclinic β- Ga_2O_3. *Phys Rev Mater* 2022, 6 (5), 1–11.
10. Duan, J. et al. Enabling propagation of anisotropic polaritons along forbidden directions via a topological transition. *Sci. Adv*. **7**, eabf2690 (2021).
11. Passler, N. et al. Hyperbolic Shear Polaritons in Low-Symmetry Crystals, *Nature* **602**, 595–600 (2022).
